# Survival time to Implanon discontinuation and its predictors among a cohort of Implanon users who enrolled in public hospitals of southern Ethiopia, 2021: a retrospective cohort study

**DOI:** 10.1186/s13690-022-00859-6

**Published:** 2022-03-23

**Authors:** Aklilu Habte, Merertu Wondimu, Hanan Abdulkadir

**Affiliations:** 1 School of Public Health, College of Medicine and Health Sciences, Wachemo University, Hosaena, Ethiopia; 2 School of nursing and midwifery, Faculty of health science, Institute of Health, Jimma University, Southwest, Jimma, Ethiopia; 3 School of Public Health, College of Medicine and Health Sciences, Arba Minch University, Arba Minch, Ethiopia

**Keywords:** Implanon discontinuation, Survival time, Predictors, Ethiopia

## Abstract

**Background:**

Pieces of evidence strongly indicate that providing high-quality family planning services is accompanied by an increase in contraceptive uptake and a decrease in contraception discontinuation. Contraceptive, especially Implanon discontinuation is a global issue that could be linked to a summary outcome indicator of poor family planning service quality. Although Implanon is becoming more popular among Ethiopian contraceptive users, little is known regarding the survival and predictors of discontinuation. The study aimed at exploring the survival and predictors of Implanon discontinuation among women enrolled in family planning units of Public hospitals in southern Ethiopia, 2021.

**Methods:**

A hospital-based retrospective cohort study was conducted among Implanon users who enrolled in family planning units of Public Hospitals. Five years of medical records, from January 1, 2016, to December 30, 2020, were reviewed. A total of 502 women were selected by using a random sampling technique. A standardized abstraction tool was used to collect data from medical records and registration books. The data were entered into Epidata Version 3.1 and then exported to STATA 14 for analysis. The median was calculated in the case of survival time. Across covariates, the Kaplan Meier survival curve was used to estimate time to Implanon discontinuation. To identify statistically significant predictors of Implanon discontinuation, a multivariable Cox proportional hazard model was fitted.

**Results:**

The incidence rate of Implanon discontinuation was 1.87(95% CI = 1.63, 2.15) per 100 person-months of observation. The overall estimated survival probability at the end of 24 and 36 months was 67.4% (95%CI, 62.5, 71.8) and 25.9% (95%CI, 18.4, 34.1) months respectively. Residence [AHR = 1.50; 95%CI: 1.09, 2.08], parity [AHR = 2.02; 95%CI: 1.65, 3.67], pre-insertion counselling [AHR = 2.41; 95%CI: 1.72, 3.70], experiencing heavy vaginal bleeding [AHR = 3.91; 95%CI: 2.67, 5.32], post-insertion follow up [AHR = 3.15; 95%CI:2.11, 4.75] were identified as a significant predictors of Implanon discontinuation.

**Conclusion:**

The risk Implanon of discontinuation was high, especially at 24 and 36 months. In family planning service delivery points, health care providers should pay special attention to clients who live in rural areas and do not have children. In addition, health care providers should provide pre-insertion counseling and post-insertion follow-up that focus on potential side effects. Finally, family planning units need to engage in early side effect management and reassurance to mitigate discontinuation.

**Supplementary Information:**

The online version contains supplementary material available at 10.1186/s13690-022-00859-6.

## Background

In 2015, 64% of married or in-union women of reproductive age group over the world used at least one method of contraception. Contraception use, on the other hand, was substantially lower in the least developed countries (40%), especially in Africa (33%) [1, 2]. Contraceptive prevalence is expected to rise from 40 to 55% in Eastern Africa between 2015 and 2030 [1]. Unwanted pregnancies account for 41% of the 208 million pregnancies that occur each year due to a lack of contraception, with approximately half of these pregnancies ending in abortion or stillbirth [3]. Contraception discontinuation is thought to be the cause of 33 million unwanted pregnancies, with grave consequences for women’s and children’s health and well-being [4–6].

Evidence strongly indicates that providing high-quality family planning services is accompanied by an increase in contraceptive uptake and a decrease in contraception discontinuation [7]. Contraceptive discontinuation is a global issue that may be linked to a lack of desire to avoid pregnancy, as well as missed opportunities to sustain contraceptive usage [8]. It’s a global issue that could be linked to a summary outcome indicator of poor family planning service quality [9, 10].

Implanon is a second-generation single-rod implantable Long-acting Reversible Contraceptive (LARC) that is implanted subdermally and provides effective contraception for up to 3 years with a clinical failure rate of less than 1% [11]. It contains 68 mg of the progestogen etonogestrel [12, 13]. It inhibits pregnancy by preventing the release of an egg from the ovary, thickening the mucus of the cervix, which may prevent sperm from reaching the egg, and altering the uterine lining [13]. It is a preferred contraceptive method for women in developing countries because of its low cost, long duration, accessibility, safety during breastfeeding, and a high chance of preserving fertility after termination [14, 15].

Although Implanon is generally well tolerated, recent studies have revealed that some women will discontinue using it for a variety of reasons [16]. Implanon discontinuation is defined as the removal of Implanon by healthcare providers in less than 3 years following insertion [17–19]. Reasons for the discontinuation can be divided into three categories: method-related (contraceptive failure), right-based (reduced need), and non-method-related (desire to become pregnant, inconvenient to use, disliked method, lack of privacy for use and, spouse disapproval [20]. Menstrual abnormalities in the form of menorrhagia or irregular vaginal bleeding, weight gain, local swelling, discomfort for no apparent cause, pain at the insertion site, and generalized weakness are all documented method-related causes for Implanon discontinuation [4, 21, 22].

The probability of Implanon discontinuation varies greatly around the world, ranging from 3 to 69% [23]. According to studies conducted in Egypt, Nigeria, and Kenya, 13.5, 19.0, and 30.0% of Implanon users, respectively, discontinued using it within the first year [24–26]. According to South African Family Practice, Implanon discontinuation was up to 43% of women before completion of the 3 years [27]. Age, prior contraceptive use, prior use of Implanon, husband’s educational level, and the existence of side effects were all independent predictors of discontinuation [17, 24, 25].

The Ethiopian Federal Ministry of Health (FMOH) initiated an Implanon scale-up program through the Integrated Family Health Program (IFHP) in 2009, intending to increase community access to and use of Implanon by allowing health extension workers to provide Implanon insertion services [28, 29]. Implanon utilization among married or in-union women grew from 3.4% in 2011 to 8% in 2016, indicating that the scale-up program was successful in reaching communities [30]. Despite this significant improvement, Implanon discontinuation is becoming unacceptably high in the country, with a discontinuation rate of 11% [30]. According to a study conducted in the Tigray region of northern Ethiopia, 16% of women discontinued using Implanon within the 1st year [31]. In a related study done in the Amhara region, 46.5% of women discontinued using Implanon at 3rd year [32].

To reduce the prevalence of unmet contraception needs, researchers need to investigate the rate to which and the reasons why contraceptive users become non-users [6]. Rather than striving to enroll a large number of acceptors at once, programs should focus on a small number of annual acceptors and focus on providing quality care to improve their satisfaction which reduces discontinuation [33]. Although Implanon is becoming more known among contraceptive users, little is known regarding the rates and reasons for discontinuation by using relatively strong study designs [34]. Those studies in Ethiopia were mostly focused on assessing early discontinuation in the first year, with the overall time to discontinuation not being evaluated rigorously. Furthermore, those studies were done based on a community-based, and they might fail to indicate the exact day of discontinuation due to recall and social desirability bias. Hence, this study aimed at determining the time to Implanon discontinuation and its predictors among users in public hospitals of southern Ethiopia. This study also quantified the rate of discontinuation across months and years. The study’s findings will help health care providers, local program planners, and other stakeholders involved in family planning and maternal health develop new initiatives to reduce Implanon discontinuation.

## Methods and materials

### Study setting

Guraghe zone is one of 13 zones of the Southern Nation Nationality and People Region (SNNPR), with a population of 1,835,110 people, 19.84% of whom are women of reproductive age (15–49 years old). Wolkite, the zone’s capital, is located 158 km south of Addis Ababa, the capital city of Ethiopia, and 337 km south of Hawassa (capital of SNNPR). Regarding health facilities, there were 74 health centers, 6 hospitals, and 444 health posts (unpublished data) [35].

### Study design and period

A retrospective cohort study was conducted in five public hospitals, based on a five-year medical record review of a cohort of Implanon users who enrolled from January 1, 2016, to December 30, 2020. The study was conducted from January 1–30, 2021.

### The population of the study

#### Source population

The source population for this study was all Implanon users who enrolled in family planning units of Public Hospitals in the Guraghe zone.

#### Study population

The study population for the study was those Implanon users who enrolled in the selected public hospitals of Guraghe zone.

#### Inclusion criteria

All the records of the Implanon users enrolled from January 1, 2016, to December 31, 2020, at the selected public hospitals of the Zone were included in the study.

#### Exclusion criteria

Those clients with incomplete records were excluded from the study.

#### Sample size determination

The sample size was calculated using the double population formula via stat calc menu of Epi info version 7 by considering the following assumptions: 95% CI; 80% power; 1:1 ratio of unexposed to exposed; the anticipated proportion of Implanon discontinuation among women with poor counseling (p1, Exposed) = 25.95%; Implanon discontinuation among women who got adequate counseling (p2, unexposed) = 14.97%; adjusted hazard ratio (AHR) =1.81 [34]. The sample size for the study was determined to be 458 based on the assumptions above. After allowing for a 10% nonresponse rate, the final sample size for the study was 502.

#### Sampling procedures

**Fig. 1:**
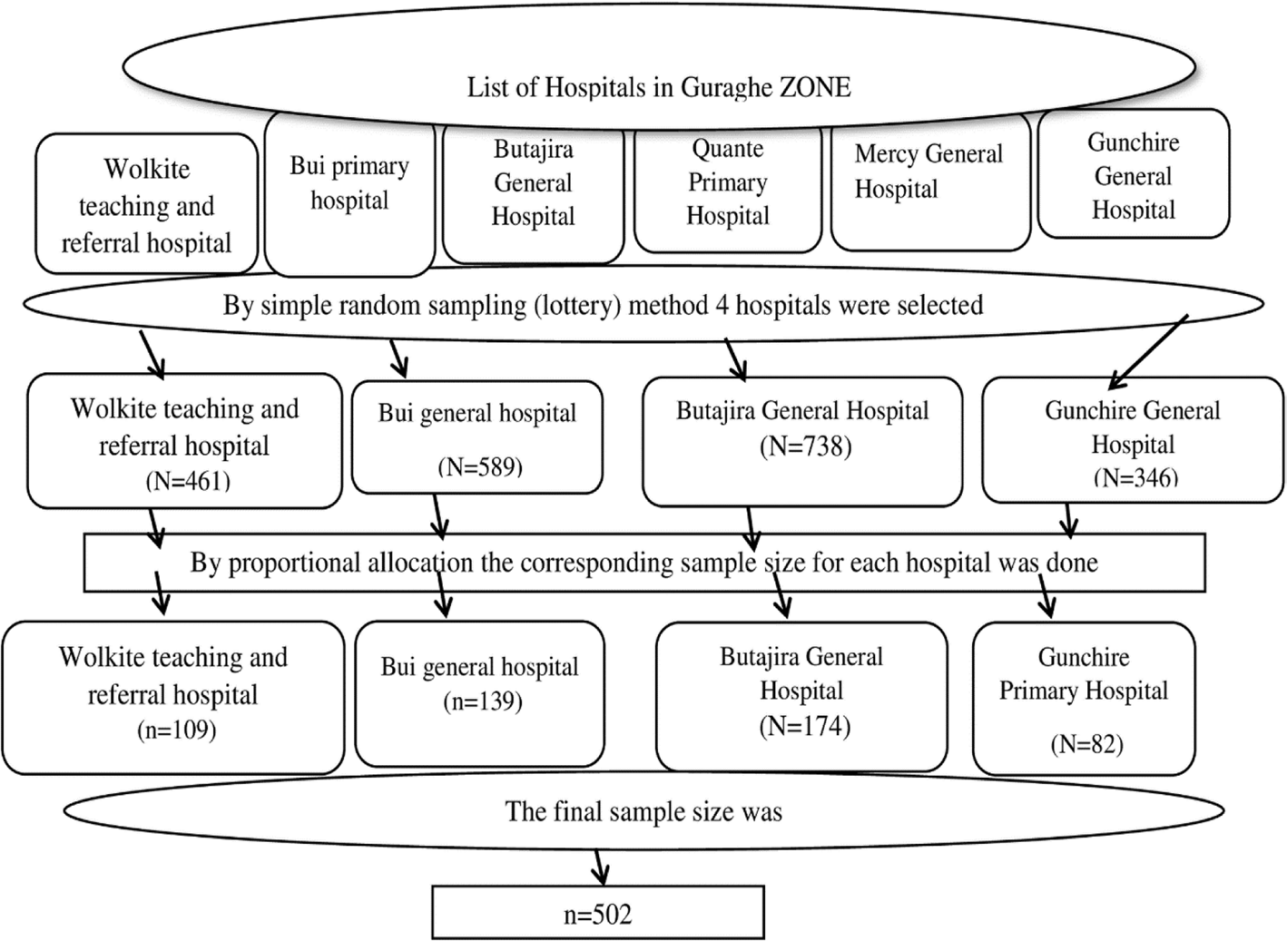
Sampling procedure for accessing study participants in Guraghe zone, southern Ethiopia, 2021

Four hospitals were selected at random (by Lottery method) among six hospitals in the zone. Then, based on the number of eligible study participants, the sample size was proportionally allocated to the selected health facilities. The study participants were chosen using a random sampling technique from 2134 records obtained from the hospitals’ Family Planning registry **(**Fig. 1**).** Due to missing variables of interest, such as individual and obstetric factors, a total of 210 records were excluded.

#### Data collection tool, methods, and personnel

A data abstraction checklist was developed by reviewing different literature in the area of interst [31, 34]. Data from the family planning service registration, Implanon removal record book and clients’ medical records were also used to develop a data abstraction checklist. During the whole data collection period, eight midwives with obstetric care experience (two per hospital) collected the data under the supervision of four public health officers. From the cohort’s medical records, variables such as age, parity, desire for more children at baseline, educational status, and marital status, past contraceptive use, side effects, and reasons for discontinuing Implanon were extracted. At the end of each day, the principal investigator cleaned, reviewed, coded, and archived all the collected data.

#### Data quality management

Before, during, and after data collection, the quality of the data was ensured. A data abstraction tool was developed by using the Federal Ministry of Health’s family planning registration book, long-acting family planning discontinuation registry, and client medical record folders. A pretest was conducted on 5% of records at Worabe Comprehensive Hospital, and possible adjustments were made based on the result. Both data collectors and supervisors got a one-day training. Before data entry, the extracted data were checked for completeness and consistency.

#### Variables of the study

The outcome variable was survival time to Implanon discontinuation which was measured in months from the date of insertion to the date of discontinuation (event). Those clients that were lost to follow-up, transferred out, or continued Implanon beyond December 30, 2020, were censored.

Predictor variables were: Age, marital status, residence, and ethnicity were sociodemographic variables. Parity (number of alive children), previous history of contraceptive use, the type of contraceptive method used, having a diagnosed hypertension, HIV test result, having counseling before insertion, facing prolonged vaginal bleeding, and having post-insertion follow up.

Implanon discontinuation (Event): Removal of Implanon by healthcare providers in less than 3 years following insertion [17–19].

#### Data analysis

The data were cleaned, coded, and entered into Epi Data version 3.1, and then exported to STATA version 14. Descriptive statistics such as mean with standard deviation and median with interquartile range have been computed to summarize the cohort’s characteristics. Pearson’s Chi-square was used to examine the relationship between categorical variables and Implanon discontinuation. The time that each participant contributed to the study was calculated by months from the date of Implanon insertion to the date of censored or discontinuation. Kaplan–Meier was used to estimate the survival probability of the cohort across a category of each predictor and to assess the overall survival at 6, 12, 24, and 36 months. A log-rank test of equality of surviving was also conducted to check for differences in the risk of discontinuation among the categories of a variable, i.e. checking whether survival probabilities are statistically significant.

Cox proportional hazards regression model was fitted to identify the predictors of time-to-discontinuation and to calculate the hazard ratios (HR). To ensure that essential predictor variables are retained, a Bivariable Cox proportional hazard Regression analysis was done first, followed by a forward variable selection using AIC (Akaike Information Criteria) with a liberal *p*-value of less than 0.1. The net effect of each explanatory variable on time to Implanon discontinuation was determined using multivariable Cox proportional hazards regression at the 0.05 level of significance. The strength of the association and statistical significance was assessed using the crude hazard ratio (CHR) and adjusted hazard ratio (AHR), with the accompanying 95% confidence interval and *P*-value. The variance inflation factor (VIF > 10) was used to check for multicollinearity between the explanatory variables, and no multicollinearity was found.

## Results

### Sociodemographic characteristics of the study participants

Among 2134 women who were enrolled from January 1, 2016, to December 30, 2020, 502 women were followed. Nearly three-fifths (58.8%) of study participants have resided in the urban areas and they accounted for 107(52.2%) of the discontinuation. The mean (±SD) age of the study subjects was 29.63 ± 5.04 years. The age group 30–34 years accounted for 205 (40.8%) of the cohort, and 94 (45.9%) of them were Implanon discontinuers (Table 1).

**Table 1:** Socio-demographic and economic characteristics of Implanon users enrolled in Family planning units of public hospitals in Guraghe zone, southern Ethiopia, 2021

Variable categories	Total = 502	Discontinued = 205	Censored = 297	Test statistics
	n (%)	n (%)	n (%)	
Age of mother in years(*n* = 502)				
< 20	46(9.2)	12(5.9)	34(11.4)	χ2 = 15.246 *P* = 0.004
20–24	36(7.2)	15(7.3)	21(7.1)	
25–29	141(28.1)	45(21.9)	96(32.3)	
30–34	205(40.8)	94(45.9)	111(33.4)	
35+	74(14.7)	39(19.0)	35(11.8)	
Marital status				χ2 = 1.864 *P* = 0.189
In marital union	433(86.3)	182(88.8)	251(84.5)	
Not in marital relation	69(13.7)	23(11.2)	46(15.5)	
Ethnicity				χ2 = 0.507 *P* = 0.776
Guraghe	417(83.1)	173(84.4)	244(82.1)	
Amhara	65(12.9)	25(12.2)	40(13.5)	
Others*	20(4.0)	7(3.4)	13(4.4)	
Residence				χ2 = 6.172 *P* = 0.016
Urban	295(58.8)	107(52.2)	188(63.3)	
Rural	207(41.2)	98(47.8)	109(36.7)	

*Others: Oromo, Hadiya

### Obstetric, medical, and maternal health service-related characteristics of respondents

At the time of insertion, 265 (52.8%) of the cohort were multipara (having 2–4 children). There was a significant difference in Implanon discontinuation between nulliparous and grand multiparous women (χ2 = 49.077, *P* < 0.001). Eighty-eight (17.5%) of the cohort had previously had an abortion. More than one-fifth of the cohort, 114 (22.7%), experienced heavy and protracted vaginal bleeding, and 104 (91.2%) of them discontinued using the Implanon (Table 2).

**Table 2:** Obstetric and maternal health service-related characteristics of Implanon users enrolled in Family planning units of public hospitals in Guraghe zone, southern Ethiopia, 2021

Variable categories	Total =502	Discontinued =205	Censored =297	Test statistics
	n (%)	n (%)	n (%)	
Parity (*n =* 502)				
0(Nulliparous)	29(57.8)	24(11.7)	5(1.7)	χ2 = 49.077 *P* < 0.001
1(Primiparous)	92(18.3)	57(27.8)	35(11.8)	
2–4(Multiparous)	265(52.8)	88(42.9)	177(59.6)	
≥ 5(Grand multiparous)	116(23.1)	36(17.6)	80(26.9)	
Previous history of abortion				
Yes	88(17.5)	42(20.5)	46(15.5)	χ2 = 2.097 *P* = 0.148
No	414(82.5)	163(79.5)	251(84.5)	
Heavy prolonged vaginal bleeding				
Yes	114(22.7)	104(50.7)	10(3.4)	χ2 = 155.20 *P* < 0.001
No	388(67.3)	101(49.3)	287(96.6)	
Weight gain				
Yes	57(11.4)	34(16.6)	23(7.7)	χ2 = 9.419 *P* = 0.002
No	445(88.6)	171(83.4)	274(92.3)	
Previous history of receiving ANC				
Yes	431(85.9)	171()	260(87.6)	χ2 = 1.702 *P* = 0.196
No	71(14.1)	34()	37(12.4)	
Previous history of receiving skilled delivery				
Yes	408(81.2)	162(79.1)	246(82.8)	χ2 = 1.153 *P* = 0.283
No	94(18.7)	43(20.9)	51(17.2)	

### Contraceptive service-related characteristics of the respondents

In terms of contraceptive use, the majority of the cohort (443, 88.2%) had previously used at least one type of contraception (repeat acceptors). Injectable contraceptives accounted for 178 (40.2%) of all contraceptive methods used by women, while oral contraceptives accounted for 116. (26.2%). The majority of study participants, 441 (87.8%), received pre-insertion counseling. Before insertion, all cohorts were offered HIV testing and counseling, and the majority, 429 (85.5%), took up that offer. Of those who received HIV testing and counseling, 18(4.2%) were positive for HIV, and all of them were linked to HART clinics (Table 3).

**Table 3:** Contraceptive service-related characteristics of Implanon users who enrolled in Family planning units of public hospitals in Guraghe zone, southern Ethiopia, 2021

Variable categories	Total =502	Discontinued =205	Censored =297	Test statistics
	n (%)	n (%)	n (%)	
Contraceptive history				
New	59(11.8)	54(26.3)	5(1.7)	χ2 = 71.100
Repeat	443(88.2)	151(73.7)	292(98.3)	*P* < 0.001
Types of contraceptives previously used (*n* = 443)				
Oral contraceptives	116(26.2)	46(25.4)	70(26.7)	χ2 = 13.676
Injectable	178(40.2)	77(42.5)	101(38.6)	*P* = 0.008
Implants	98(22.1)	30(16.6)	68(25.9)	
IUCD	27(6.1)	11(6.1)	16(6.1)	
Condom	24(5.4)	17(9.4)	7(2.7)	
Provider of FP				
Midwives	241(48.1)	101(49.3)	140(47.1)	χ2 = 3.807 *P =* 0.283
Public health officers	164(32.6)	72(35.1)	92(31.0)	
Nurses	60(11.9)	18(8.8)	42(14.1)	
General practitioners	37(7.4)	14(6.8)	23(7.8)	
Offered with pre-insertion counseling				
Yes	441(87.8)	151(73.7)	290(97.6)	χ2 = 65.360
No	61(12.2)	54(26.3)	7(23.5)	*P* < 0.001
Had Post-insertion follow up				
Yes	219(43.6)	34(16.6)	185(62.3)	χ2 = 103.014
No	283(56.4)	171(83.4)	112(37.7)	*P* < 0.001
Having a diagnosed HTN				
Yes	70(13.9)	36(17.6)	34(11.4)	χ2 = 3.777
No	432(86.1)	169(82.4)	263(88.6)	*P =* 0.052
HIV test performed				
Yes	429(85.5)	177(86.3)	252(84.8)	χ2 = 0.218
No	73(14.5)	28(13.7)	45(15.2)	*P* = 0.641
HIV test result(*N =* 429)				
Positive	18(4.2)	11(6.2)	7(2.8)	χ2 = 3.055
Negative	411(95.8)	166(93.8)	245(97.2)	*P* = 0.080
Got HIV specific FP counseling				
Yes	18(3.6)	11(5.4)	7(23.6)	χ2 = 3.055
No	484(96.4)	194(94.6)	290(97.6)	*P =* 0.080

### Overall survival status of Implanon users

**Fig. 2:**
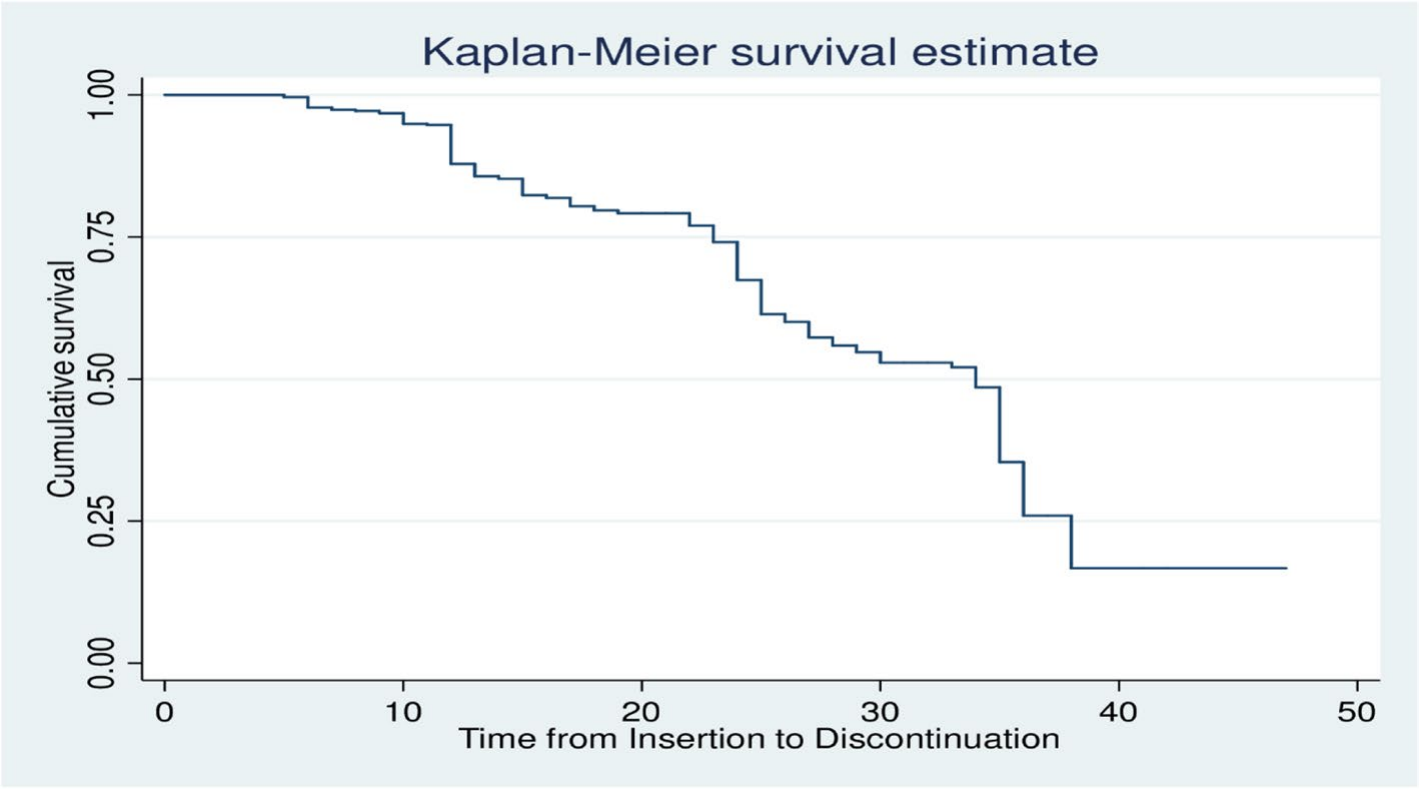
The overall Kaplan-Meier survival estimate curve of Implanon users who enrolled in Family Planning units of public hospitals in southern Ethiopia, 2021

Out of 502 women who were followed for a total of 60 months, 205(40.5%) of them have discontinued Implanon. The remaining 208(41.4%), 46(9.2%), 39(7.8%), and 4(0.8%) were current users, lost to follow up, transferred out, and died respectively. The total person-months contributed by the study participants were 10,943. The overall incidence density of discontinuation among the cohort was 1.87(95%CI = 1.63–2.15) per 100 person-months of observation or 22.4(95%CI = 19.56–25.8) per 100 person-years of observation (PYO). According to the Kaplan Meier survival estimation, the overall median survival time was 34 months (95% CI: 31.84, 36.16) (Fig. 2). The incidence density of Implanon discontinuation among rural and urban women was 2.25and 1.62 per 100 PM, respectively (Table 4).

**Table 4:** Incidence Density of Implanon discontinuation among Strata of Categorical Variables of women who enrolled in family planning units of Public hospitals in Guraghe zone, southern Ethiopia, 2021

Variable categories	Number of women discontinued (*n* = 205)	Person time in months (PM)	Incidence Density (per 100 PM)
Age of mother in years			
< 20	12(5.9)	1606	2.42
20–24	15(7.3)	4434	2.11
25–29	45(21.9)	3053	1.47
30–34	94(45.9)	827	2.11
35+	39(19.0)	1023	2.42
Marital status			
Not in marital relation	23(11.2)	9498	1.91
In marital union	182(88.8)	1444	1.59
Residence			
Urban	107(52.2)	6603	1.62
Rural	98(47.8)	4340	2.25
Parity			
≥ 5(Grand multiparous)	36(17.6)	2586	1.39
2–4(Multiparous)	88(42.9)	5789	1.52
1(Primiparous)	57(27.8)	1982	2.87
0(Nulliparous)	24(11.7)	586	4.09
Contraceptive history			
Repeat	163(73.7)	9676	1.68
New	42(26.3)	1267	3.30
Types of contraceptives previously used			
Condom	17(9.4)	423	4.01
IUCD	17(9.4)	596	2.85
Oral contraceptives	46(25.4)	2564	1.79
Injectable	71(39.2)	3908	1.81
Implants	30(16.6)	2109	1.42
Heavy and prolonged vaginal bleeding			
No	101(49.3)	8823	1.14
Yes	104(50.7)	2120	4.90
Pre-insertion counseling			
Yes	151(73.7)	9681	1.55
No	54(26.3)	1262	4.27
Had Post-insertion follow up			
Yes	34(16.6)	4892	0.7
No	171(83.4)	6051	2.8
Having a diagnosed HTN			
No	169(82.4)	9535	1.772
Yes	36(17.6)	1408	2.55
Previous history of abortion			
No	163(79.5)	8891	1.83
Yes	42(20.5)	2052	2.04

**Fig. 3:**
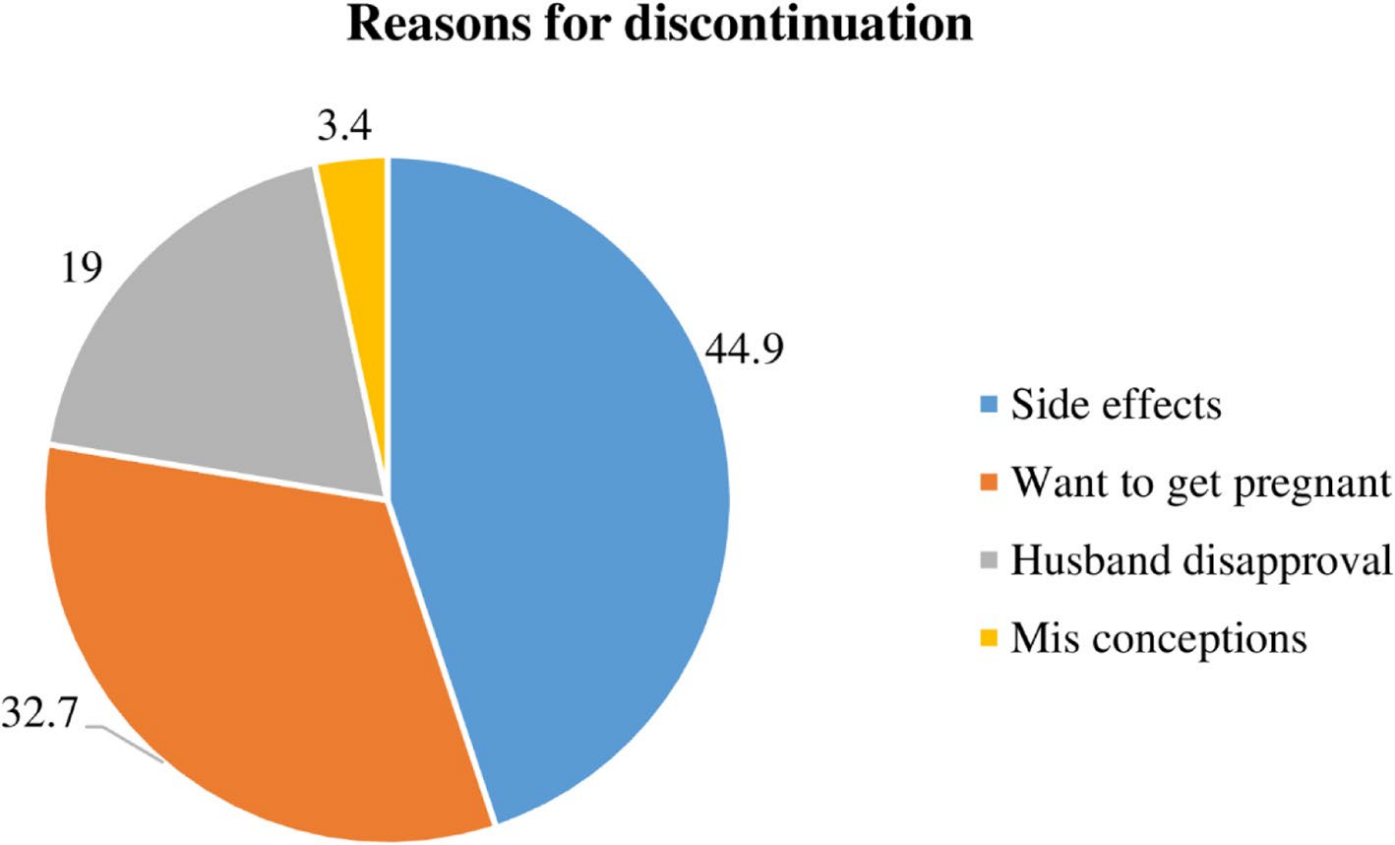
The reasons for discontinuation among Implanon users in public hospitals of southern Ethiopia, 2021

There was a substantial increase in discontinuation in the late follow-up period, notably after 12 months. The overall estimated survival probability was 97.8% (95%CI: 96.6, 98.8), 87.9% (95%CI: 84.62, 90.5), 67.4% (95%CI: 62.5, 71.8) and 25.9% (95%CI: 18.4, 34.1) at the end of 6, 12, 24 and 36 months respectively. There were no women who discontinued using the Implanon within the first 4 months, and just two women discontinued at the fifth month. Only 54 (26.3%) of women who discontinued using Implanon got post-removal contraception. The commonest reason for discontinuation was side effects, by 92 (44.9%) of the women (Fig. 3).

### The Kaplan Meier estimates of median survival time to Implanon discontinuation among covariates

The mean survival time was determined in this study since the maximum observation period was censored. According to the Kaplan–Meier survival function, women who had previously taken any contraceptives (Repeat acceptors) had a greater survival time than women who had never used contraceptives (new acceptors) (31.49 months, 95%CI = 29.63–33.34). this difference was statistically significant at a *p*-value< 0.001. The mean survival time for women living in urban areas was 31.49 months (95% CI = 29.63, 33.34) (*P* < 0.05) (Table 5).

**Table 5:** The Kaplan Meier estimates of mean survival time for women who used Implanon according to different characteristics during five years follow-up in public hospitals of Guraghe zone, southern Ethiopia, 2021

Variable Categories	Mean survival time in months (95%CI)	Log-rank Estimates
Age of mother in years		
< 20	31.87(29.81,33.94)	χ2 = 21.863
20–24	29.23(25.88,32.59)	*p*-value = 0.018
25–29	33.02(29.97,36.06)	
30–34	28.14(26.44,29.83)	
35+	26.01(23.36,28.66)	
Marital status		
In marital union	29.95(28.51,31.39)	χ2 = 0.140
Not in marital relation	28.2(25.70,30.83)	*p*-value = 0.018
Residence		
Urban	31.49(29.63,33.34)	χ2 = 7.777
Rural	27.33(25.58,29.07)	*p*-value = 0.005
Parity		
0(Nulliparous)	21.67(17.91,25.43)	χ2 = 33.543,
1(Primiparous)	25.29(23.12,27.46)	*p*-value< 0.001
2–4(Multiparous)	32.42(30.22,34.63)	
≥ 5(Grand multiparous)	30.95(29.09,32.81)	
Previous history of receiving ANC		
Yes	30.29(28.78,31.80)	χ2 = 1.508
No	27.51(24.65,30.36)	*p*-value = 0.259
Previous history of receiving skilled delivery		
Yes	28.46(28.48,31.63)	χ2 = 0.111
No	30.06(26.03,30.89)	*p*-value = 0.739
Contraceptive history		
New	21.16(18.73,23.60)	χ2 = 57.797
Repeat	31.89(30.28,33.49)	*p*-value< 0.00
Types of contraceptives previously used		
Implants	31.27(28.63,33.92)	χ2 = 19.199
Injectable	30.39(28.01,32.78)	*p*-value = 0.004
Oral contraceptives	29.51(27.41,31.61)	
IUCD	25.72(21.51,29.93)	
Condom	20.36(16.03,24.69)	
Previous history of abortion		
Yes	29.08(26.81,31.35)	χ2 = 0.012
No	30.47(28.82,32.12)	*p*-value = 0.914
Having a diagnosed HTN		
Yes	24.91(22.44,27.38)	χ2 = 7.571
No	30.63(29.14,32.11)	*p*-value = 0.006
HIV test and counseling offered		
Yes	29.87(28.40,31.34)	χ2 = 0.837
No	29.63(26.82,32.44)	*p*-value = 0.360
HIV test performed		
Yes	29.96(28.43,31.49)	χ2 = 0.627
No	29.48(27.03,31.94)	*p-*value = 0.428
HIV test result		
Positive	22.27(17.59,26.94)	χ2 = 7.746
Negative	30.29(28.73,31.87)	*p-*value = 0.005
Got pre-insertion counseling		
Yes	31.92(30.30,33.54)	χ2 = 47.107
No	21.56(19.13,24.02)	*p-*value< 0.001
Service provider		
Midwives	30.08(28.22,31.95)	χ2 = 0.25 *p-*value = 0.615
Other health care providers	28.84(27.26,30.42)	
Heavy prolonged vaginal bleeding		
Yes	19.32(17.67,20.98)	χ2 = 165.515 *p-*value< 0.001
No	34.43(32.68,36.18)	
Having post-insertion follow-up insertion		
Yes	35.53(33.76,37.29)	χ2 = 71.293, *p-*value< 0.001
No	25.84(24.36,27.33)	

**Fig. 4:**
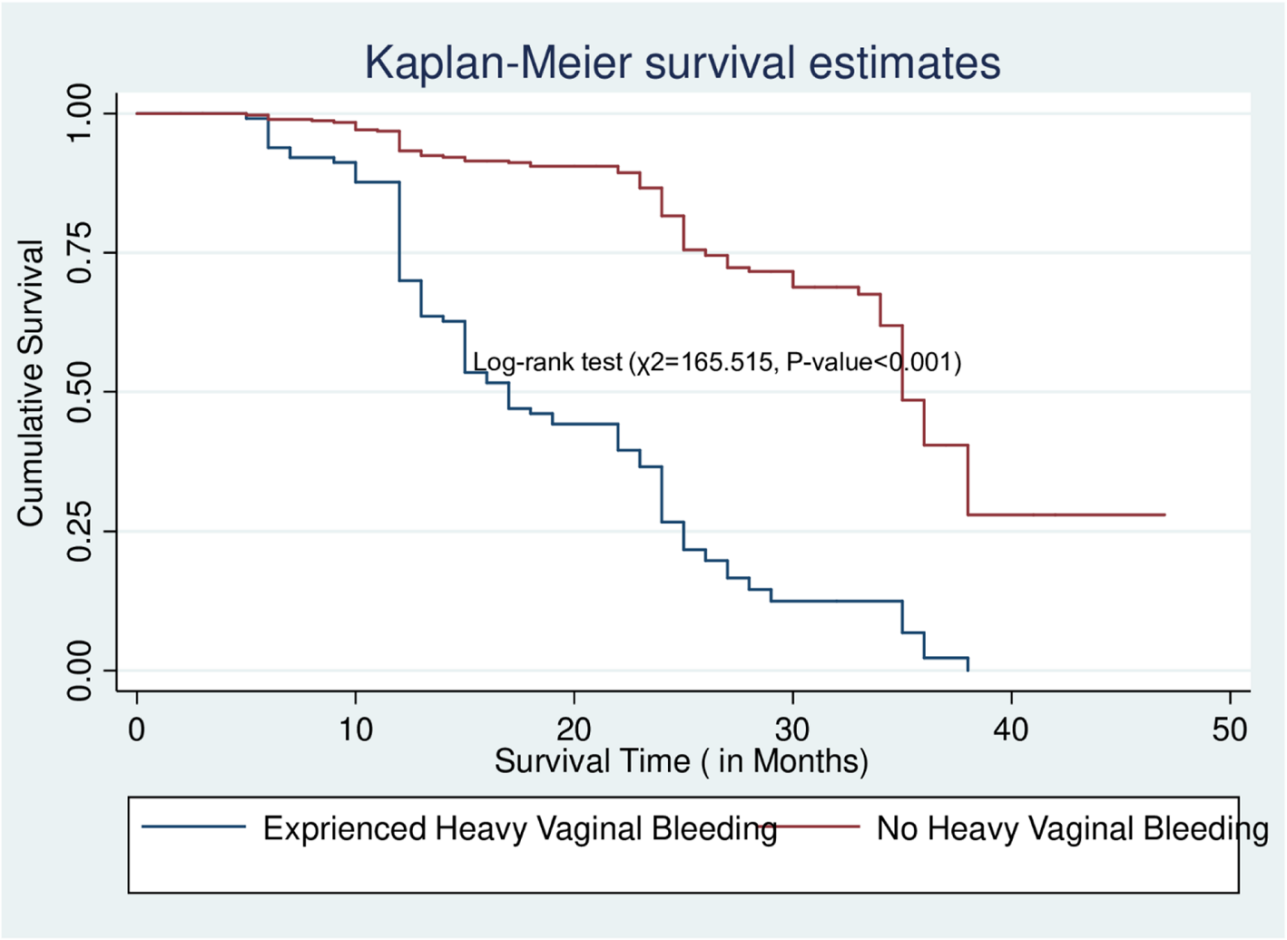
The Kaplan–Meier survival curves that compare survival time of Implanon Users by experiencing prolonged heavy vaginal bleeding in Public hospitals of Guraghe zone, Southern Ethiopia, 2021

Women who did not have heavy, persistent vaginal bleeding had a longer survival time than those who did (34.43 months, 95% CI = 32.68–36.18), which was statistically significant at *p*-value< 0.001(Fig. 4).

**Fig. 5:**
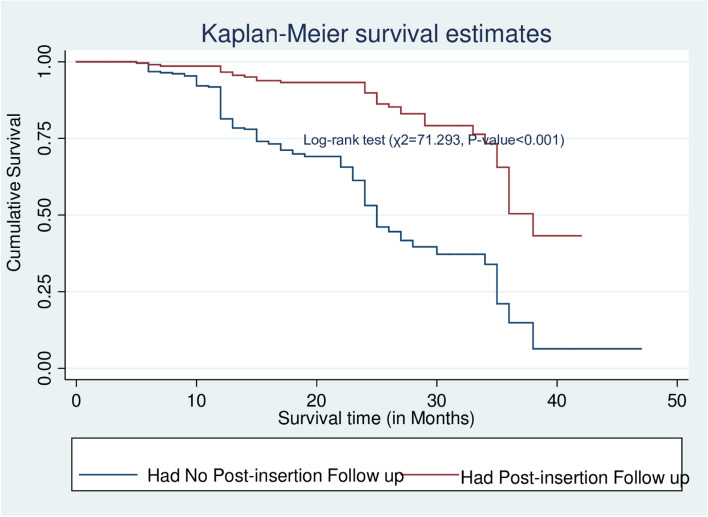
The Kaplan–Meier survival curves compare survival time of Implanon Users by having a post-insertion follow up in Public hospitals of Guraghe zone, Southern Ethiopia, 2021

The mean survival time for women who received post-Insertion follow-up was longer (35.53 months, 95%CI = 33.76–37.29) than for those who did not (25.84 months, 95%CI = 24.36–27.3), according to the Kaplan–Meier graph and the Log-rank test (Fig. 5).

### Predictors of time to Implanon discontinuation

In the bivariable Cox Proportional Hazard regression model eleven variables namely, maternal age, residence, parity, being a new acceptor, pre-insertion counseling, post-insertion follow up, the type of contraceptive previously used, having a prolonged and heavy bleeding, weight gain, diagnosed with hypertension, previous history of abortion, were associated with Implanon discontinuation (*P* < 0.25). After controlling for confounding with a multivariable Cox Proportional Hazard Regression, six variables were identified as significant predictors of Implanon discontinuation: residence, parity, counseling before insertion, post-insertion follow up, heavy and prolonged vaginal bleeding, and previous uptake of Implanon.

When compared to women who did not live in rural areas, the hazard of discontinuing Implanon was 1.5 times higher among rural women [AHR = 1.50; 95%CI: 1.09, 2.08]. Parity was also found to be a predictor of Implanon discontinuation. Women who had no child (nulliparous) had a two-fold higher likelihood of discontinuance than those who had five or more children [AHR = 2.02; 95%CI: 1.65, 3.67]. Women who did not get pre-insertion counseling had a 2.4 times higher chance of discontinuing their Implanon than those who did [AHR = 2.41; 95%CI: 1.72, 3.70]. In terms of side effects, women who experienced significant and/or persistent vaginal bleeding were 3.9 times more likely to discontinue the Implanon than their counterparts [AHR = 3.91; 95%CI: 2.67, 5.32]. Women without post-insertion follow-up had a 3.15 times higher risk of discontinuance than those with recorded follow-up schedules [AHR = 3.15; 95%CI:2.11, 4.75]. When comparing women who previously used Implanon as a contraceptive technique to those who used a condom as a contraceptive method, those who previously used Implants had a 53% lower likelihood of discontinuation [AHR = 0.47; 95%CI: 0.25,0.87] (Table 6).

**Table 6:** Bi-variable and multivariable Cox proportional hazard regression for predictors of Implanon discontinuation among women enrolled in Family planning units of public hospitals in Guraghe zone, southern Ethiopia, 2021

Variable Categories	Total [n(%)]	Discontinued [n(%)]	CHR(95%CI)	AHR(95%CI)	*p-*value
Age of mother in years					
< 20	46(9.2)	12(5.9)	1	1	1
20–24	36(7.2)	15(7.3)	1.40(0.66,3.00)	0.84(0.37,1.93)	0.683
25–29	141(28.1)	45(21.9)	1.24(0.65,2.34)	1.01(0.52,1.94)	0.986
30–34	205(40.8)	94(45.9)	1.68(0.92,3.07)*	1.54(0.82,2.89)	0.178
35+	74(14.7)	39(19.0)	1.94(1.01,3.71)*	1.43(0.70,2.90)	0.325
Marital status					
Not in marital relation	69(13.7)	23(11.2)	1		
In marital union	433(86.3)	182(88.8)	1.08(0.70,1.68)		
Residence					
Urban	295(58.8)	107(52.2)	1	1	1
Rural	207(41.2)	98(47.8)	1.45(1.10,1.91)*	1.50(1.09.2.08)**	0.014
Parity					
≥ 5(Grand multiparous)	116(23.1)	36(17.6)	1		
2–4(Multiparous)	265(52.8)	88(42.9)	1.09(0.74,1.62)	0.90(0.58,1.40)	0.639
1(Primiparous)	92(18.3)	57(27.8)	2.01(1.32,3.05)*	1.51(0.75,2.79)	0.240
0(Nulliparous)	29(57.8)	24(11.7)	2.89(1.72,4.85)*	2.02(1.65,3.67)	0.021
Ever got ANC service					
Yes	431(85.9)	171(83.4)	1	1	1
No	71(14.1)	34(16.6)	1.25(0.86,1.81)		
Ever got skilled delivery					
Yes	408(81.2)	162(79.1)	1		
No	94(18.7)	43(20.9)	1.06(0.75,1.48)		
Contraceptive history					
Repeat	443(88.2)	163(79.5)	1	1	
New	59(11.8)	42(20.5)	1.97(1.40,2.77)*	1.79(0.89,2.96)	0.061
Types of contraceptives previously used (*n =* 443)					
Condom	24(5.4)	17(9.4)	1	1	
IUCD	27(6.1)	17(9.4)	0.58(0.29,1.14)	0.56(0.26,1.21)	0.141
Oral contraceptives	98(22.1)	46(25.4)	0.37(0.21,0.65)*	0.58(0.32,1.06)	0.078
Injectable	178(40.2)	71(39.2)	0.38(0.22,0.64)*	0.532(0.27,1.05)	0.069
Implants	98(22.1)	30(16.6)	0.30(0.17,0.55)	0.47(0.25,0.87)**	0.016
Previous history of abortion					
No	414(82.5)	163(79.5)	1	1	
Yes	88(17.5)	42(20.5)	1.02(0.72,1.43)*	1.59(0.83,2.44)	
Having a diagnosed HTN					
No	432(86.1)	169(82.4)	1	1	
Yes	70(13.9)	36(17.6)	1.63(1.14,2.35)*	1.59(0.93,2.44)	0.055
Counseled before insertion					
Yes	441(87.8)	151(73.7)	1	1	
No	61(12.2)	54(26.3)	2.73(2.00,3.73)*	2.4(1.72,3.70)**	0.021
Heavy and prolonged vaginal bleeding					
No	388(67.3)	101(49.3)	1	1	1
Yes	114(22.7)	104(50.7)	4.88(3.70,6.44)*	3.91(2.67,5.32)**	< 0.001
Weight gain					
No	445(88.6)	171(83.4)	1	1	
Yes	57(11.4)	34(16.6)	1.36(0.91,2.05)*	1.152(0.74,1.885)	
Having post-insertion follow up					
Yes	219(43.6)	34(16.6)	1	1	1
No	283(56.4)	171(83.4)	4.09(2.83,5.91)*	3.15(2.11,4.71)*	< 0.001

Key: 1: Reference category; *AHR* Adjusted hazard ratio, *CHR* Crude Hazard ratio, *statistically significant at *p-*value< 0.1, ** statistically significant at *p*-value < 0.05

## Discussion

This retrospective cohort study was aimed to assess the survival and predictors of Implanon discontinuation among women who enrolled in the family planning units of Public Hospitals in southern Ethiopia. At the end of follow-up, 205(40.8%) women were discontinued and 297 were censored. This finding resulted in a cumulative incidence of removal of 22.4(95%CI = 19.56–25.8) per 100 person-years of observation which is higher than studies conducted in the Democratic Republic of Congo, Nigeria, and Thailand with an overall incidence rate of 11.52, 19 and 2.3 and per 100 person-years of observation respectively [34, 36, 37]. This could be related to differences in the study area, as more than half of the women in this study lived in rural settings, where there may be a lack of awareness of potential side effects due to a lack of media and post-insertion follow-up, which could result to early Implanon removal. As a result, interventions that delay the premature discontinuation of Implanon must be emphasized by local governmental and non-governmental groups working on family planning.

In the current study the discontinuation rate was 12.1% in 12 months, which was higher than studies conducted in Democratic Republic of Congo (8.4%) [34], Nigeria (8.1%) [25], Kenya (8.0%) [26], Senegal (6.3%) [38]. But lower than studies conducted in northern Ethiopia, Mekele city (15.7%) [39] and Debremarkos Town (23.9%) [32]. The discontinuation rate at 24 months in the current study was 32.6%, which was higher than studies conducted in Northern Ethiopia (19.3%) [39]. Democratic Republic of Congo (20.0%) [34], and Nigeria (19.3%) [25] The discontinuation rate in the current study at 3rd year was 74.9% and is higher than studies conducted in Debre Markos town (46.5%) and Mekele city (62%), both in Northern Ethiopia [32, 39]. The disparity in discontinuation rates could be due to differences in the study area, study design, sociodemographic characteristics of the study participants, pre-insertion counseling, and post-insertion follow-up. In general, the risk of discontinuance rose as time went on, which could be owing to the appearance of some side effects and their linked difficulties, women’s desire for conception, and the occurrence of husbands’ influence.

Parity was found to be a strong predictor of discontinuation. The risk of discontinuance was two times higher among women who had no child (nulliparous) compared to those who had 5 or more children. This finding is supported by studies conducted in Bangladesh [8], the Democratic Republic of Congo [34], and northern Ethiopia [17, 32]. A possible justification could be that women who have no living children want to have children and hence have a higher likelihood to discontinue using Implanon. If the side effects are perceived as a threat to their future fertility, childless women may be more likely to stop [17]. Additionally, those nulliparous women may be in a relationship at the time of insertion, and once married, they may have a strong desire and pressure to have a child, which may lead to discontinuance. Those grand multiparous women, on the other hand, may be more motivated not to discontinue Implanon early since they are more likely to have achieved their intended family size and may be more driven to avoid another pregnancy [34]. This could have implications for early pregnancy, and health care providers working at Family Planning service delivery points should pay special attention to those clients through robust counseling. However, the present findings on parity differ from a study conducted in Ethiopia, which found that for every extra child, the likelihood of discontinuing modern contraception dropped by 12% [40].

The Hazard of discontinuation among women with no pre-insertion counseling was 2.4 times higher as compared to those women who got counseling. This finding was consistent with studies conducted in Ethiopia [17, 32, 41], Jordan [42], and the Democratic Republic of Congo [34]. This implies that a lack of effective side effect counseling may lead to a negative attitude toward methods once clients have unpleasant effects, resulting in early removal and might be limiting their usage in the future [43]. On the other hand, women who receive adequate counseling before insertion may be more aware of potential side effects and how to deal with them, which is one of the most effective ways of retaining the Implanon than removing it earlier [28, 44]. Therefore, before providing the service, health care providers should carefully assess the clients depending on their reproductive strategy as a spacer or limiter.

Women without documented post-insertion follow-up had a 3.15 times higher risk of discontinuing Implanon than women with documented follow-up schedules. This conclusion was supported by studies conducted in northern Ethiopia (Amhara and Tigri regions) [17, 31] and southern Ethiopia [41]. This could be due to a sense of safety engendered by caring professionals and prompt treatment of potential side effects [43]. Furthermore, if the users have a complaint at the follow-up visit, the care providers could provide an appropriate solution for the challenges and health problems that they had while using Implanon, which may help to avoid discontinuation. Hence, to increase the longevity of the Implanon, health care providers in family planning service delivery points must place a due emphasis on follow-up afterward insertion.

Those who had previously used Implants had a 53% lower likelihood of discontinuing Implanon than those who had previously used a condom as a contraceptive method. A study conducted in Egypt agreed with this finding [24]. This could be because women who have previously used implants are more aware of and familiar with some of the side effects, and are therefore less frustrated once they happened, resulting in the use of the method for a long time. As a result, service providers must make a concerted effort to provide new Implanon users with pre-insertion counseling and post-insertion follow-up.

The presence of prolonged/heavy vaginal bleeding has also been shown to have a significant effect on the incidence of discontinuation. This was in line with studies conducted in northern Ethiopia [17, 32], Southern Ethiopia [41], Democratic Republic of Congo [34], and Egypt [24]. This could be due to an unanticipated shift in the monthly bleeding pattern caused by the Implanon, making ordinary tasks difficult and leading to discontinuation. This finding has many implications. Service providers should be aware that even minor bleeding can cause women to get anxious and cease using the method if they receive no or inadequate counseling [34]. As a result, health care providers should address possible bleeding with caution and encourage women to return if they have any concerns. During the follow-up period, special attention should be paid to the intensity and length of vaginal bleeding to assess the severity of the problem and rule out other reasons such as infection, drug interactions, and gynecological pathology.

### Strength and limitation of the study

To our knowledge, this study may be the first of its kind at the national level in assessing the incidence and predictors of discontinuation among Implanon users by using a fairly strong study design. As a result, these findings may have relevant programmatic input for policymakers, healthcare providers, and other stakeholders participating in FP programming as an input to intervene in discontinuation by understanding the possible predictors. Because records with missing information or charts that were lost for certain patients were excluded, selection bias may have occurred. On the other hand, because the data is gathered through record review, the incompleteness of information and the reliability of the recorded data, such as excessive vaginal bleeding, remains a major concern.

## Conclusion

The risk Implanon of discontinuation was high, especially at 24 and 36 months. Residence, parity, pre-insertion counseling, Post-insertion follow-up, heavy and prolonged vaginal bleeding, and previous uptake of Implanon were identified as significant predictors of Implanon discontinuation. In family planning service delivery points, health care providers should pay special attention to clients who live in rural areas and do not have children. In addition, health care providers should provide pre-insertion counseling and post-insertion follow-up that focus on potential side effects. In addition, family planning departments must engage in early side effect treatment and reassurance to mitigate discontinuation.

## Supplementary Information

**Additional file 1.**

**Additional file 2.**

## Data Availability

The data used to support the findings of the current study can be obtained from the corresponding author on reasonable request via akliluhabte57@gmail.com.

## Abbreviations

AHR: Adjusted Hazard Ratio
CHR: Crude Hazard Ratio
EDHS: Ethiopian Demographic Health Survey
FMOH: Federal Ministry of Health
VCT: Voluntary HIV/AIDS Counselling, and Testing
WHO: World Health Organization
