# Supplementary material for: Survival time to Implanon discontinuation and its predictors among a cohort of Implanon users who enrolled in public hospitals of southern Ethiopia, 2021: a retrospective cohort study
Source: Arch Public Health. 2022 Mar 23;80:89. doi: 10.1186/s13690-022-00859-6 (PMC8941807; doi:10.1186/s13690-022-00859-6)
Supplement: Supplementary file 2 — Additional file 2. [file 13690_2022_859_MOESM2_ESM.docx]

## Annex2. Data abstraction tool for the study conducted on “Survival time to Implanon discontinuation and its predictors among a cohort of Implanon users who enrolled in public Hospitals of Southern Ethiopia, 2021: A retrospective cohort study”

| **S No** | | **Questions** | | | | **Response** | | | **Code** |
| --- | --- | --- | --- | --- | --- | --- | --- | --- | --- |
| **Part I: Identifications** | | | | | | | | |  |
| 101 | | Hospital Name | | | | | 1. Butajira tertiary hospital 2. Wolkite teaching and referral hospital 3. Bui primary hospital 4. Gunchire Primary Hospital | |  |
| 102 | | Hospital code | | | | | _________________ | |  |
| 103 | | Date form filled to start | | | | | ____/___/____ | |  |
| 104 | | Maternity record number/Medical Reg. Number | | | | | _________________ | |  |
| **Part II Socio demographic and economic characteristics of respondents** | | | | | | | | |  |
| 201 | Place of residence | | | 1. Urban 2. Rural | | | | |  |
| 202 | Maternal age in years | | | 1. [___________] | | | | |  |
| 203 | marital status | | | 1. Married 2. Divorced 3. Unmarried/single 4. Widowed | | | | |  |
| 204 | What is your ethnicity? | | | 1. Guraghe 2. Amhara 3. Oromo 4. Hadiya 5. Wolaita 6. Others | | | | |  |
| **Part III: Obstetric And Maternal Health Service Related Characteristics** | | | | | | | | |  |
| 301 | | | Number of children alive | |  | | | |  |
| 302 | | | Ever had abortion | | 1. Yes 2. No | | | |  |
| 303 | | | Previous history of receiving ANC | | 1. Yes 2. No | | | |  |
| 304 | | | Previous history of receiving skilled delivery | | 1. Yes 2. No | | | |  |
| 305 | | | Contraceptive history | | NEW REPEAT | | | |  |
| 306 | | | Types of contraceptives previously used(for Repeat) | | 1. Oral contraceptives  2. Injectable  3. Implants  4. IUCD  5. Condom | | | |  |
| 307 | | | Provider of FP | | 1. Midwives  2. Public health officers  3. Nurses  4. General practitioners  5. Others | | | |  |
| 308 | | | Offered with pre-insertion counseling | | 1. Yes 2. No | | | |  |
| 309 | | | Had Post-insertion follow up | | 1. Yes 2. No | | | |  |
| 310 | | | Had a diagnosed HTN | | 1. Yes 2. No | | | |  |
| 311 | | | Heavy prolonged vaginal bleeding | | 1. Yes 2. No | | | |  |
| 312 | | | Weight gain | | 1. Yes 2. No | | | |  |
| 313 | | | HIV test offered | | 1. Yes 2. No | | | |  |
| 314 | | | HIV test performed | | 1. Yes 2. No | | | |  |
| 315 | | | HIV test result | | 1. Positive 2. Negative | | | |  |
| 316 | | | Date of Insertion(DD/MM/YY) | | _____/____/____ | | | |  |
| 317 | | | End date (DD/MM/YY) | | Removal | | | _____/____/____ |  |
|  |  |  |  |  | Currently used | | | _____/____/____ |  |
|  |  |  |  |  | Transferred out | | | _____/____/____ |  |
|  |  |  |  |  | Loss to follow up | | | _____/____/____ |  |
|  |  |  |  |  | Death | | | _____/____/____ |  |

**THANK YOU!!!!!**
